# The changing landscape of automated insulin delivery in the management of type 1 diabetes

**DOI:** 10.1530/EC-23-0132

**Published:** 2023-07-31

**Authors:** Rama Lakshman, Charlotte Boughton, Roman Hovorka

**Affiliations:** 1 Wellcome-MRC Institute of Metabolic Science, Addenbrooke's Hospital, Cambridge, UK; 2 Cambridge University Hospitals NHS Foundation Trust, Wolfson Diabetes and Endocrine Clinic, Cambridge, UK

**Keywords:** automated insulin delivery, artificial pancreas, closed-loop systems, type 1 diabetes

## Abstract

Automated insulin delivery systems, also known as closed-loop or ‘artificial pancreas’ systems, are transforming the management of type 1 diabetes. These systems consist of an algorithm which responds to real-time glucose sensor levels by automatically modulating insulin delivery through an insulin pump. We review the rapidly changing landscape of automated insulin-delivery systems over recent decades, from initial prototypes to the different hybrid closed-loop systems commercially available today. We discuss the growing body of clinical trials and real-world evidence demonstrating their glycaemic and psychosocial benefits. We also address future directions in automated insulin delivery such as dual-hormone systems and adjunct therapy as well as the challenges around ensuring equitable access to closed-loop technology.

## Introduction

Over nine million people worldwide live with type 1 diabetes (T1D) (1). In this condition, immune-mediated destruction of pancreatic beta-cells leads to insulin deficiency and resultant hyperglycaemia. The management of T1D necessitates lifelong administration of exogenous insulin at appropriate doses to keep blood glucose levels within the target range.

Intensifying insulin therapy to minimise hyperglycaemia is important to reduce the risk of long-term macrovascular and microvascular complications (2). Optimal glycaemic control is often limited by the risk of hypoglycaemia, and is made more challenging because insulin needs vary considerably day to day (3). A minority of people with T1D currently achieve the recommended glycaemic targets (4), and the high management burden associated with the condition can lead to reduced quality of life, burnout, diabetes distress, and depression (5).

There have been rapid advancements in diabetes technology since the discovery of insulin a century ago. Continuous subcutaneous insulin infusion (CSII) pumps were first developed in the 1970s and have since markedly reduced in size and increased in capability. Continuous glucose monitoring (CGM) devices, measuring real-time interstitial glucose concentration, have been available since 1999 and have steadily improved in accuracy and reliability (6). Insulin pump therapy is associated with improved glycaemic control and reduced hypoglycaemia compared with multiple daily insulin injections (7), and CGM is associated with improved glucose control and reduced hypoglycaemia compared to fingerstick capillary glucose monitoring (8, 9, 10, 11). Neither insulin pump therapy, CGM, or the use of both together as sensor-augmented pump (SAP) therapy reduces management burden (12); requiring frequent user input to respond to glucose values and manually adjust insulin doses.

**Figure 1:**
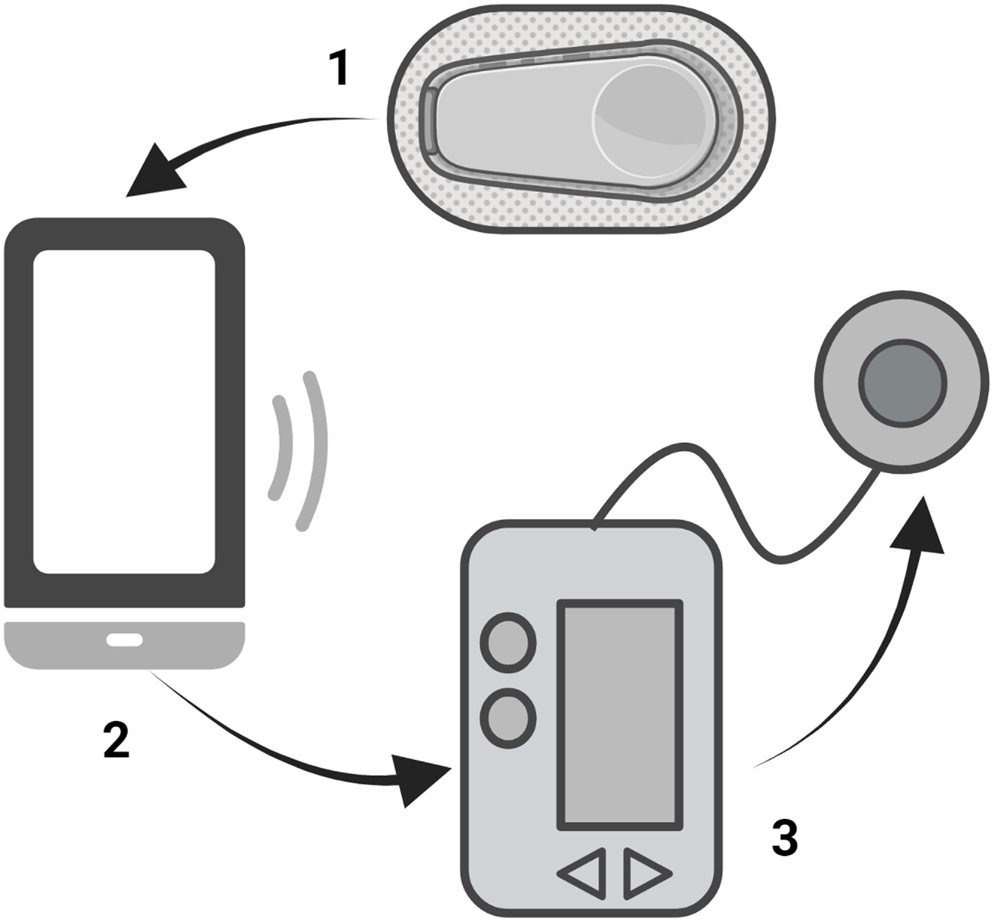
A closed-loop automated insulin delivery system comprising (1) a subcutaneous glucose monitor which communicates real-time glucose levels to (2) a device hosting the control algorithm which responds by regularly adjusting insulin delivery via (3) a subcutaneous insulin pump. Communication between systems is wireless. (Created with BioRender.com).

Automated insulin delivery (AID) systems address this issue by linking the glucose sensor and insulin pump via an algorithm which automatically adjusts insulin delivery in response to glucose levels (Fig. 1). These closed-loop systems, sometimes referred to as an ‘artificial pancreas, have the potential to not only improve glycaemic control but also reduce diabetes burden and improve quality of life. Here, we review the changing landscape of AID: from initial development to current practice and future directions.

## Methods

A literature search of PubMed and Google Scholar was conducted using keywords ‘closed-loop’, ‘automated insulin delivery’, ‘artificial pancreas’ and ‘type 1 diabetes’. The search was restricted to papers published in English over the last 15 years. Additional studies were identified from cited articles.

## Past: the development of automated insulin delivery systems

### Early intravenous systems

**Figure 2:**
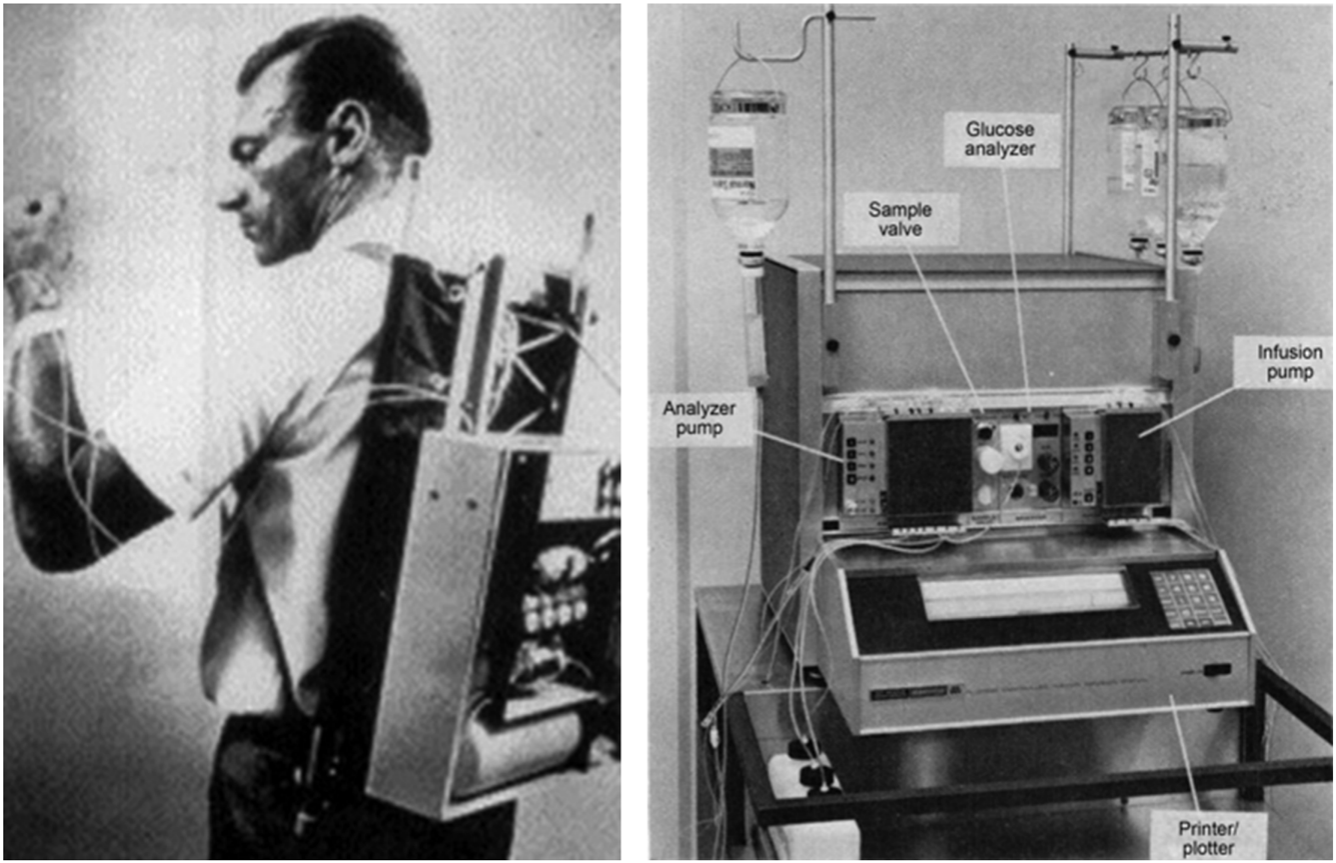
Early automated insulin delivery systems (A) The first insulin pump, developed by Kadish. (B) The Biostator computer-based glucose-controlled insulin infusion system. Reproduced with permission from Alsaleh FM, Smith FJ, Keady S & Taylor KM, ’Insulin pumps: from inception to the present and toward the future’, *Journal of Clinical Pharmacy and Therapeutics,* copyright 2010 John Wiley and Sons (93).

The first intravenous AID system was developed in 1963 by Arnold Kadish (13). It comprised of an intravenous glucose monitor and two intravenous syringe pumps: a pump delivering insulin which was activated when glucose level rose above the higher threshold and a pump delivering either glucose or glucagon which was activated when glucose fell below the lower threshold. It never made it to market due to its impracticality, being the size of an army backpack (Fig. 2A).

The first commercial AID system was the Biostator (Miles Laboratories, Elkhart, IN, USA), developed in the 1970s by Pfeiffer and colleagues (14). It consisted of a pump which controlled continuous venous blood withdrawal, a continuous blood glucose analyser, a computer algorithm to calculate the amount of insulin or dextrose to be infused, and an infusion pump for intravenous insulin/ dextrose delivery (Fig. 2B). Its size and complexity meant the Biostator was limited to inpatient use, but it was extensively used in research in the late 20th century (15).

### First-generation automated insulin delivery systems

**Figure 3:**
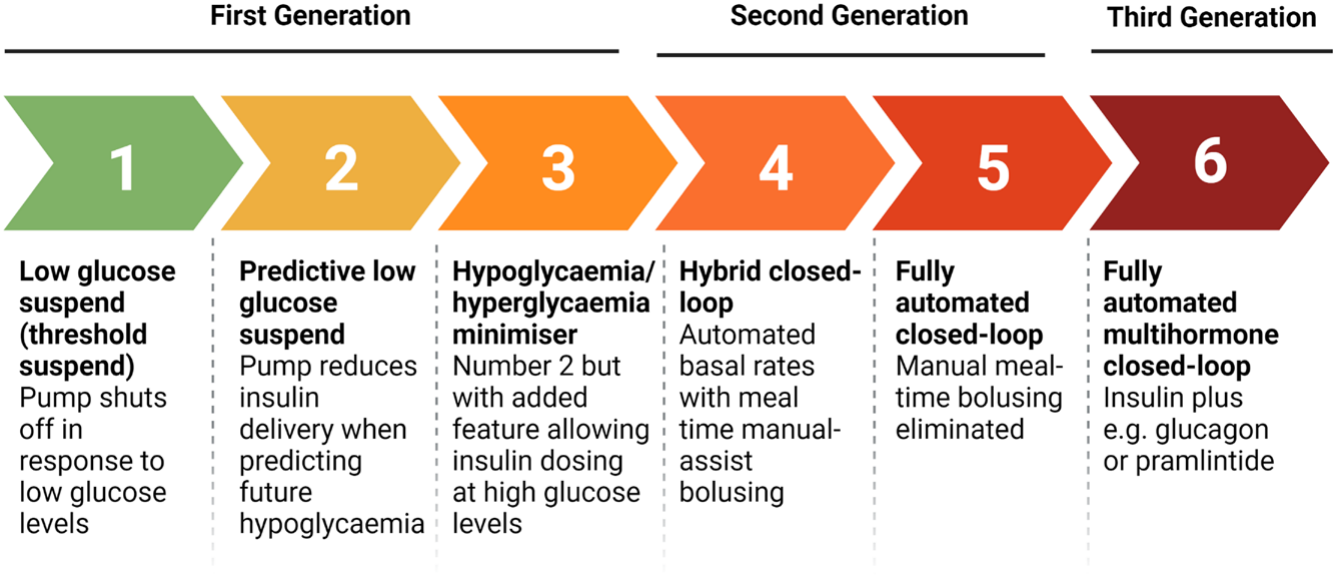
The six developmental stages of artificial pancreas device systems as originally described by JDRF (https://www.jdrf.org/blog/2011/02/09/artificial-pancreas-and-fda-the-latest/). (Created with BioRender.com).

Numerous increasingly small and reliable CSII pumps and interstitial CGM systems were developed in the 2000s, making subcutaneous-subcutaneous AID systems a feasible therapy option. In 2005, JDRF established the Artificial Pancreas Project with the aim of promoting the development of AID technologies (15). JDRF defined six categories of AID technology, based on the level of automation involved (Fig. 3).

The simplest stage of automation was a low glucose suspend (threshold suspend) system, where the pump automatically suspends insulin delivery when sensor glucose drops below a pre-specified value. The first low glucose suspend system, the MiniMed Paradigm Veo /530G (Medtronic, Northridge, CA, USA), was released in 2009. The next stage up was predictive low glucose suspend (PLGS) systems, which include an algorithm that predicts future hypoglycaemia and pre-emptively reduces insulin delivery. PLGS technology first became commercially available in 2015 with the MiniMed 640G (Medtronic) and then in 2018 with the t:slim X2 Basal-IQ (Tandem, San Diego, CA, USA). Compared to non-automated pump and sensor systems, both LGS and PLGS are associated with a significant reduction in hypoglycaemia (16, 17), although PLGS did increase hyperglycaemia in a paediatric population (18).

Increasing automation was achieved by adding to the PLGS system a feature to automatically give a small correction bolus when glucose was predicted to increase above a pre-specified threshold. These predictive hyperglycaemia and hypoglycaemia minimisation systems were associated with improved overnight glycaemic control in both children and adults with TID (19, 20), but never made it to commercial products.

### Control algorithms for automated insulin delivery

Subsequently, several research groups began developing more complex control algorithms to automatically adjust insulin delivery every 5–10 min based on real-time sensor glucose levels, with the aim of more closely replicating normal pancreatic physiology. There are three main types of control algorithms that have been utilised in these closed-loop systems: proportional-integral-derivative (PID) controllers, model predictive control (MPC) controllers, and fuzzy logic controllers. PID controllers modify insulin rates by evaluating glucose excursions from three perspectives: deviation from target glucose (proportional component), area under the curve between measured and target glucose level (integral component), and rate of change of measured glucose levels (derivative component) (21). MPC algorithms predict future glycaemic excursions and adjust insulin delivery based on inputs including sensor glucose levels and insulin boluses given, simultaneously considering insulin absorption delays, active insulin, and diurnal and post-prandial variability in glucose levels (22). The fuzzy logic approach is less commonly used and involves modulating insulin delivery based on rules which reflect the reasoning of experienced diabetes practitioners.

### Development of hybrid closed-loop systems

The most advanced AID systems currently available are hybrid closed-loop (HCL) systems, where the control algorithm adjusts the basal insulin rate, but users must administer prandial insulin boluses for optimal control. Over the past decade and a half, HCL systems have undergone extensive testing: from safety studies in controlled laboratory settings (23, 24, 25), to transitional supervised outpatient settings (26, 27, 28), and finally to overnight and day-and-night studies under home free-living conditions (29, 30, 31, 32, 33). In 2016, the MiniMed 670G (Medtronic) became the first commercially available HCL system, with the pivotal trial in 124 participants over 3 months showing a significantly increased time in range compared to baseline (34). Since then, there has been exponential growth in the field, with six HCL systems now approved for use in people with TID between North America and Europe.

## Present: current landscape in automated insulin delivery

### Commercial hybrid closed-loop systems

**Figure 4:**
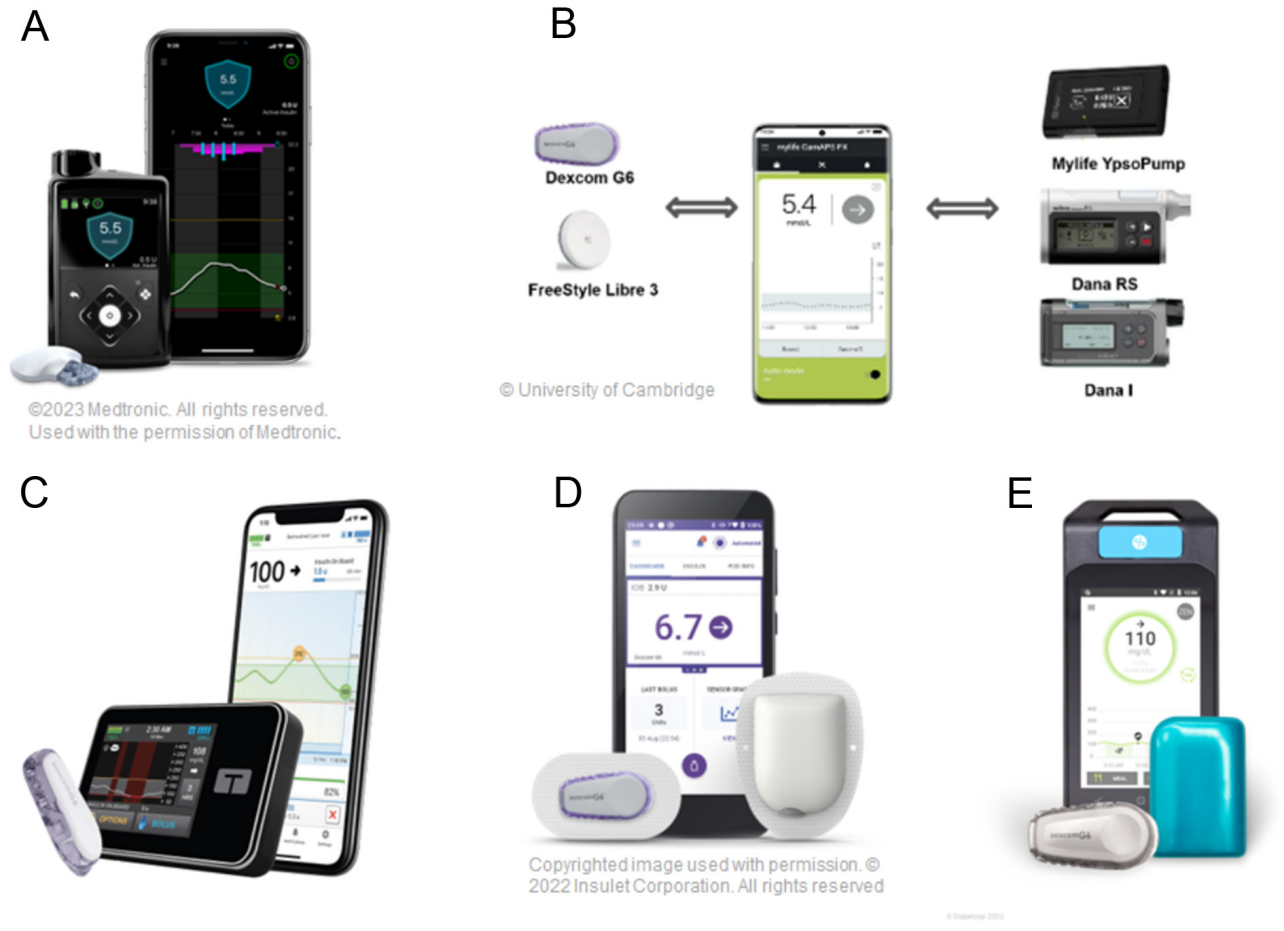
Commercially available hybrid closed-loop systems. (A) MiniMed TM 780G with Guardian 4 sensor; ©2023 Medtronic. All rights reserved. Used with the permission of Medtronic. (B) CamAPS FX algorithm on a smartphone with Dana or YpsoPump and Dexcom G6 or Freestyle Libre 3 sensor; CamAPS FX copyright University of Cambridge 2023. (C) Tandem t:slim X2 pump with Dexcom G6 sensor; copyright 2023 Tandem Diabetes Care. (D) Insulet Omnipod 5 with patch pump and Dexcom G6 sensor; © 2023 Insulet Corporation. (E) Diabeloop DBLG1 algorithm with Kaleido patch-pump and Dexcom G6 sensor; © 2023 Diabeloop SA.

Today, five manufacturers have licenced HCL systems for people with T1D: Medtronic MiniMed 670G/770G/780G, CamDiab (Cambridge, UK) CamAPS FX, Tandem (San Diego, CA, USA) Control-IQ, Insulet (Acton, MA, USA) Omnipod 5, and Diabeloop (Grenoble, Rhone-Alpes, France) DBLG1 (Fig. 4). All these systems follow the same basic principles but differ in terms of the algorithm, hardware, and functionality (Table 1).

**Table 1:** Comparison of commercially available automated insulin delivery systems.

	Medtronic 670G/770G/780G	CamAPS FX	Tandem t:slim X2 with Control-IQ	Diabeloop DBLG1	Insulet Omnipod 5
License	670G, 770G: CE label and FDA label	CE label	CE label and FDA label	CE label	CE label and FDA label
	780G: CE label	Licensed in Australia			
Licenced indications	≥2 years (770G), ≥7 years (670G, 780G)	≥1 year and ≥10kg	≥6 years and ≥25kg	≥18 years	≥2years
	TDD 8–250 U/day	TDD 5–350 U/day	TDD 10–100 U/day	8–90 U/day	≥5 U/day
	Pregnancy excluded	Pregnancy included	Pregnancy excluded	Pregnancy excluded	Pregnancy excluded
Licensed insulin	Rapid acting	Rapid acting and ultra-rapid acting	Rapid acting	Rapid acting	Rapid acting
Compatible pump	MiniMed 670G	YpsoPump	t:slim X2	Accu-chek Insight Kaleido	Omnipod 5
	MiniMed 770G	Dana I			
	MiniMed 780G	Dana RS			
Compatible CGM	Guardian 3 (670G, 770G)	Dexcom G6	Dexcom G6	Dexcom G6	Dexcom G6
	Guardian 4 (780G)	Freestyle Libre 3			
Mobile control	View app on phone (780G)	Full phone control (only Android)	Phone bolusing (iOS and Android) (US only)	No	Full phone control (only Android)
Algorithm location	Pump integrated	App-based	Pump-integrated	Dedicated handset	Pod-integrated
Type of algorithm	PID with insulin feedback (670G, 770G)	MPC	MPC	MPC	MPC
	Additional model based auto-corrections (780G)				
Target	6.7 mmol/L (670G, 770G)	Personalised target 4.4–11.0 mmol/L; up to 48-time blocks per day	Target 30-min predicted range of 6.25–8.9 mmol/L; corrections down to 6.1 mmol/L	Personalised target 5.6–7.2 mmol/L	Personalised target 6.1–8.3 mmol/L; up to 8-time blocks per day
	Adjustable 5.5, 6.1 or 6.7 mmol/L (780G)				
Other modes	Activity mode: target of 8.3 mmol/L and no autocorrections	Ease-off mode: target 7 mmol/L, less insulin delivery ~−35%	Sleep Activity: target 6.25–6.7 mmol/L basal rate modulation only;	Activity mode: target +3.8 mmol/L and less aggressive algorithm	Activity mode: target 8.3 mmol/L restricted insulin delivery
		Boost mode: insulin delivery ~+35%, more responsive algorithm	Exercise activity: target 7.8–8.9 mmol/L	Zen mode: target +0.5–2.2 mmol/L and less aggressive algorithm	
Pre-set basal insulin rates influence AID	No	No	Yes (users can set multiple basal rates and different basal profiles)	No	No (informs AID for first 48 h)
Bolus correction delivery	Automated correction boluses – up to 12 per hour (780G)	Automated corrections via intensive basal rate adjustments	Automated basal rate adjustment every 5 min and automated correction boluses up to 1 per hour	Automated correction boluses	Automated corrections via intensive basal rate adjustments
	Optional user-initiated correction boluses	Optional user-initiated correction boluses	Optional user-initiated correction boluses	Optional user-initiated correction boluses	Optional user-initiated correction boluses
Active insulin time	Adjustable 2–8 h	Automatically adjusted based on adaptive learning	Fixed 5 h	Automatically adjusted based on adaptive learning	Adjustable 2–6 hs
Adaptive learning	Yes – TDD estimated fasting glucose and plasma insulin	Yes – TDD, diurnal, meals	Yes – TDD tracked over time	Yes – TDD diurnal, meals	Yes – TDD
Automatic data upload for remote monitoring	No – 670G	Yes – Diasend/Glooko once hourly	Hourly via t:connect mobile app (USA only)	Glucose data via Dexcom follow	Yes – Glooko once hourly
	Yes – Carelink (770G/ 780G)	Real-time monitoring with Companion app and SMS	Glucose data via Dexcom follow		

CE mark, Conformity Europeenne mark; CGM, continuous glucose monitoring; FDA, United States Food and Drug Administration; MPC, model predictive control; PID, proportional integral derivative; TDD, total daily dose of insulin.

### DIY automated insulin delivery systems

The do-it-yourself (DIY) closed-loop movement began in 2013, when a community of people with T1D and their families began collaborating online to develop their own artificial pancreas systems (APS), behind the hashtag #WeAreNotWaiting. These DIY systems connect commercially available insulin pumps and CGMs to an open-source algorithm, which does not undergo any regulatory oversight or approval. The three main systems, Loop, OpenAPS, and AndroidAPS, were used by around 1500 people with TID in 2019 (35). Even now HCL therapy is commercially available, and these DIY systems remain appealing to those who have the confidence and skills to maintain them, due to lower costs and increased customisability (22). The not-for-profit company Tidepool (Palo Alto, CA, USA) developed a commercial version of the iOS app Loop (Tidepool Loop), which recently became the first DIY algorithm to be approved by the FDA (https://www.tidepool.org/blog/tidepool-loop-has-received-fda-clearance). The company are currently working on partnerships with CGM and pump manufacturers.

### Glycaemic outcomes

All commercially available HCL systems have been shown to be safe and efficacious for people living with T1D. An initial meta-analysis of 40 randomised controlled trials (RCTs) of several different AID systems in the outpatient setting demonstrated improved glycaemic control compared to control therapy: with increased percentage time spent in the target glucose range of 3.9–10.0 mmol/L (weighted mean difference +9.6 percentage points, 95% confidence interval (CI) +7.5 to +11.7%), reduced hypoglycaemia <3.9 mmol/L (weighted mean difference −1.5 percentage points , 95% CI −1.9 to −1.1%), and a favourable effect on HbA1c (weighted mean difference −0.26%, 95% CI −0.38 to −0.13%) (33). A more recent network meta-analysis of ten RCTs found that closed-loop systems led to a greater time in target glucose range than any other management strategy: mean time in range was 17.9 percentage points higher when compared to multiple daily injections with capillary glucose monitoring and 8.8 percentage points higher when compared to insulin pump therapy with CGM (36).

Comparisons of efficacy between HCL systems are hampered by differences in participant baseline characteristics and study design. The only head-to-head comparison of two different HCL systems compared the Medtronic MiniMed 670G with the second-generation MiniMed 780G (37). In this multinational randomised crossover trial of 113 adolescents and young adults with T1D, the use of the MiniMed 780G led to a reduction in time spent in hyperglycaemia > 10.0 mmol/L by 3.0 percentage points (95% CI −4.0 to −2.0%), without increasing hypoglycaemia compared with the MiniMed 670G.

HCL systems have now been tested in randomised trials in vulnerable cohorts, including the extremes of ages. In a crossover trial of 37 older adults (aged 60 years or above) with T1D, the use of CamAPS FX led to an increase in time in the range of 8.6 percentage points compared to SAP, importantly with no increased risk of hypoglycaemia (38). In 74 very young children (aged 1–7 years) with T1D, CamAPS FX led to an increased time in the range of 8.7 percentage points, without increased hypoglycaemia (39). The Tandem Control-IQ system has also been evaluated in 102 very young children aged 2–6 years in a parallel design trial, with those in the HCL group having 12.4 percentage points more time in range compared to usual care (40).

Pregnancy is a challenging time for people with T1D to achieve the tighter recommended glycaemic targets. A small study of 16 pregnant women showed that day-and-night closed-loop insulin delivery was associated with significantly less hypoglycaemia and comparable glucose control compared to SAP therapy (41). Larger trials of the MiniMed 780G system (NCT04520971), CamAPS FX system (NTC04938557), and Tandem Control-IQ system (NCT04902378) in pregnancy are all currently underway.

The first randomised control of an open-source AID system (a modified version of AndroidAPS 2.8 with a standard OpenAPS 0.7.0 algorithm) was recently conducted on 97 participants over 24 weeks. Compared to SAP, the use of an open-source AID system was associated with an increase in time in the target glucose range of 14 percentage points (42).

### Psychosocial outcomes

A growing body of qualitative research on HCL systems report a number of user benefits including reassurance and reduced anxiety, improved sleep, and ‘time off’ from diabetes demands (43). While some studies report benefits in diabetes-specific quality of life measures assessed by validated questionnaires, these findings have not been consistent (44, 45, 46, 47, 48). User feedback makes it clear that the benefits of AID are balanced by challenges including variable levels of trust in the system, physical bulk of devices, alarm burden and connectivity problems (43, 46).

Arguably the greatest quality-of-life benefits of closed-loop systems have been reported in the caregivers of young children with T1D, who have the highest burden of diabetes management (49). Caregivers of very young children using the CamAPS FX system reported less anxiety knowing that the system would help keep glucose in range, better sleep, increased confidence to leave their child with others, and being able to resume normal activities including in some cases return to full-time employment (50). Similarly, the use of the Control-IQ AID system in children with TID significantly improved sleep and psychosocial measures in parent poor sleepers (51), and the use of open-source AID systems has been associated with improved quality of life and sleep in children and caregivers (52).

Lived experience has been directly compared between the MiniMed 670G and second-generation MiniMed 780G systems (53). While there was no difference in diabetes distress or hypoglycaemia confidence, the 780G system was associated with improved glucose monitoring satisfaction. The Omnipod 5 is the first commercially available tubeless on-body AID system, and data from the recent pivotal trial showed improvements in diabetes distress, hypoglycaemia confidence, and diabetes treatment satisfaction after 3 months of system use (44).

Users without previous experience of HCL can have unrealistically high expectations of the technology, with terms like ‘artificial pancreas’ and ‘closed-loop system’ being potentially misleading in suggesting that no user input is required (54). Managing expectations of HCL systems clearly at the outset is important in avoiding disappointment and promoting long-term usage and optimal outcomes (55).

### Real-world outcomes

As more people use AID systems, there is increasing real-world data on utility and glycaemic outcomes. A prospective observational study of Medtronic Minimed 670G users showed that AID utilisation correlated with improved glycaemic control, but there was a high discontinuation rate, with 33% stopping closed-loop by 12 months (56). Promisingly, real-world data on large numbers of users of the second-generation MiniMed 780G AID system (57) as well as the Tandem control-IQ (58), Diabeloop DBLG1 (59), and Loop DIY system (60) all show a median of over 80% time spent using closed-loop, and a sustained time in the range of over 70% at the end of the observation period.

Real-world data are now available across the age groups. Data from over 10,000 MiniMed 780G system users show that children aged 15 years or younger (*n* = 3211) achieve similar glycaemic outcomes to those older than 15 years (*n* = 8874), with over 75% achieving >70% time in range (61). Real-world data from 48 older adults (mean age 70 ± 4 years) showed that starting the Control-IQ AID system led to improved glycaemic control and reduced time in hypoglycaemia compared with prior therapy (62).

In the United Kingdom, National Health Service England recently conducted a real-world pilot to collect data on a range of HCL systems. Across 300 person-years of AID observations, time in range increased by 28.5 percentage points in adults with suboptimal control (HbA1c > 70 mmol/mol, 8.5%), and 14.3 percentage points in children, with a decrease in hypoglycaemia in both cohorts (63, 64).

## Future: emerging directions in automated insulin delivery

### Simplified meal announcements

All currently available HCL systems require users to count carbohydrates and manually bolus before meals for optimal glycaemic outcomes. Accurate carbohydrate counting is frequently challenging, requires a level of numeracy and literacy that can be a barrier for some, and adds significantly to the burden of day-to-day diabetes management (65).

One approach to reducing the burden of carbohydrate counting is through simplified, meal announcements. In the iLet Bionic Pancreas (Beta Bionics) algorithm, meals are announced in terms of size (usual, more, or less) relative to other meals of the same type (i.e. breakfast, lunch, dinner). In 165 children and adolescents with T1D, those randomised to use the closed-loop system increased time in range by 10 percentage points over 13 weeks compared with standard care (66). In an RCT of the MiniMed 780G system, adolescents achieved an average of 73.5% time in range with simplified meal announcements (choosing one of three personalised fixed carbohydrate amounts), though carbohydrate counting further improved outcomes to 80.3% (67).

### Fully closed-loop systems with ultra-rapid insulin

The ultimate goal for AID technology is a fully closed-loop system, where the algorithm automatically determines both basal and bolus insulin requirements, with no user input required. The main barrier to a fully closed loop is the delayed action of subcutaneously administered rapid-acting insulin analogues, which results in post-prandial glucose excursions in the absence of pre-meal boluses. New faster acting insulin analogues such as Fiasp (Novo Nordisk) and Lyumjev (Eli Lilly) are now available. While these have only shown small overall benefits over standard insulin analogues when applied in HCL systems (68, 69), there is evidence of reduced postprandial hyperglycaemia, particularly after a missed meal bolus (70). A recent randomised crossover study using AndroidAPS with Fiasp in 16 adolescents found no significant difference between fully closed-loop (with no meal announcements) and HCL glucose control over 3 days in a controlled camp setting, with time in the range of 81 and 83%, respectively (71). Longer outpatient studies comparing the CamAPS HX fully closed-loop system using ultra-rapid insulin to SAP therapy are currently underway in adults (NCT04977908) and adolescents (NCT05653050) with suboptimal glycaemic control.

### Bihormonal fully closed-loop systems

In addition to insulin, the secretion of glucagon and amylin is impaired in people with T1D (22). Both hormones are important in glycaemic control; glucagon reduces hypoglycaemia by stimulating hepatic glucose release in response to falling glucose levels, and amylin reduces post-prandial hyperglycaemia by delaying gastric emptying. An alternative approach to achieve a fully-closed loop system includes incorporating one of these hormones alongside insulin.

Glucagon and insulin dual-hormone systems, without meal announcements (72) or with simple meal announcements (73), have been found to reduce both hyperglycaemia and hypoglycaemia compared to conventional insulin pump therapy. One of these systems, Inreda (Inreda Diabetic, Goor, the Netherlands) is the first CE-marked bi-hormonal AID system and has around 125 users in the Netherlands (72). A major drawback is the unstable liquid formulation of glucagon, which requires daily replacement and the need for two separate pump systems. Chemically stable synthetic glucagon analogues, for example, dasiglucagon, have been recently developed, and preliminary results from a trial of the dual-hormone iLet system are promising (74).

Pramlintide and insulin dual-hormone systems have been found to improve post-prandial hyperglycaemia when compared to an insulin-only HCL system (75). In a supervised inpatient study, participants using a pramlintide and Fiasp fully closed-loop system spent 74.3% of time in target range, although this was still lower than with the Fiasp alone HCL system (76). Current challenges of using pramlintide in a longer-term outpatient setting include gastrointestinal side effects, and the need for a separate pramlintide infusion pump; however, an insulin-pramlintide coformulation is under development (65).

### Automated insulin delivery with adjunct therapies

Other adjunctive therapies have been evaluated to optimise glycaemic control and potentially reduce the need for carbohydrate counting. Sodium-glucose cotransporter-2 (SGLT2) inhibitors lower plasma glucose by blocking renal reabsorption and increasing the excretion of glucose in the urine. Their use in T1D is limited due to an increased risk of euglycaemic ketoacidosis (77). In outpatient crossover RCTs utilising the iPancreas AID system, 25 mg empagliflozin daily with HCL led to an increase in time in range compared to HCL alone (78), and 25 mg empagliflozin with simple meal announcements was non-inferior to HCL alone (79). However, in both studies, there was an increase in ketone levels associated with the use of empagliflozin. A follow-up outpatient randomised crossover study evaluated a ten times lower dose of empagliflozin as an adjunct to HCL in adults with T1D and suboptimal control (HbA1c 7–10.5%, and time in range <70% after 2 weeks on HCL) and found that 2.5 mg of empagliflozin increased time in range by 13 percentage points (from 59.0 to 71.6%), with no difference in mean ketone levels compared with HCL alone (80).

Glucagon-like peptide-1 (GLP-1) analogues increase satiety, slow gastric emptying, and suppress glucagon release. Small inpatient studies of GLP-1 agonists with fully closed-loop therapy seem promising (81), and a longer outpatient study looking at weekly subcutaneous semaglutide as an adjunct to closed-loop therapy is currently underway (NCT05205928).

### Advances in hardware

Pump and sensor hardware can impact the user experience of AID systems as much as the algorithm itself. The newest CGM devices do not require finger stick calibration and are rapidly decreasing in size; with the Freestyle Libre 3 CGM the size of two stacked UK pennies (https://www.freestyle.abbott/uk-en/products/freestyle-libre-3.html). Conventionally, insulin pump users have to change infusion sets every 2–3 days, but extended wear sets are now available. The Medtronic 7-day insulin infusion set has recently been shown to be safe and associated with high user satisfaction when used with an HCL system (82). Combining extended wear infusion sets with a CGM as a single device has the potential to further reduce device burden; however, the interference of nearby insulin delivery with glucose sensing continues to present a challenge (83).

Advances in hardware may also allow for additional device integration to optimise AID algorithms. Meal announcements could be simplified through the use of a smartwatch application capable of detecting eating behaviour (84), while exercise could be automatically recognised and adjusted for through the integration of heart rate, skin temperature, and accelerometer data (85). Continuous ketone monitors used in combination with AID could reduce the incidence of DKA and would be particularly useful for those taking adjunctive SGLT2 inhibitor therapy (86).

### Improved access to AID systems

Arguably far more important than technological advances in AID systems is improving access to these technologies for all those who could benefit. Closed-loop therapy is associated with significant upfront and ongoing costs compared with standard insulin therapy, and whilst health economic analyses from a range of countries are favourable (87, 88, 89), reimbursement remains varied between and within territories. A lack of equitable access to AID technology is likely to increase disparities in the management of TID; particularly as those from lower socio-economic backgrounds are more likely to have suboptimal glycaemic control (90), and therefore have the most to gain from these systems. With national and international guidance and consensus statements being updated, there is hope that reimbursement will soon be more widely available (64, 91).

Another barrier to wider access closed-loop systems is clinical inertia, linked to concerns from healthcare professionals around the additional need for staff training and user support, as well as geographical variations in technological experience (92). Healthcare systems are increasingly stretched, but manufacturers and technology experts can help by creating online resources for both professionals and system users.

## Conclusion

The landscape of AID in the management of TID has evolved rapidly over the past few decades. Initial bulky prototypes have evolved into a range of refined algorithms and compact hardware options, which are being gradually embedded into routine clinical practice. Given growing clinical trials and real-world data showing glucose control and quality of life benefits across a range of populations, it is likely that HCL therapy will become the standard of care for many people with TID in the near future. Future directions include fully closed-loop and dual-hormone systems, adjunct therapy and additional hardware integration. Equally important is ensuring access to these technologies to all those who could benefit, through equitable reimbursement strategies and healthcare provider training.

## Declaration of interest

RL declares no duality of interest associated with the present manuscript. CKB has received consultancy fees from CamDiab and speaker honoraria from Ypsomed. RH reports having received speaker honoraria from Eli Lilly, Dexcom, and Novo Nordisk, receiving consultancy fees from Abbott Diabetes Care, receiving license fees from BBraun, and being director at CamDiab.

## Funding

Work in the authors’ group is supported by the National Institute of Health Research http://dx.doi.org/10.13039/100005622 Cambridge Biomedical Research Centre, Efficacy and Mechanism Evaluation National Institute for Health Research http://dx.doi.org/10.13039/100005622, and The Leona M & Harry B Helmsley Charitable Trust.
